# Antioxidant, Antimicrobial and Tyrosinase Inhibitory Activities of Xanthones Isolated from *Artocarpus obtusus* F.M. Jarrett

**DOI:** 10.3390/molecules17056071

**Published:** 2012-05-21

**Authors:** Najihah Mohd. Hashim, Mawardi Rahmani, Gwendoline Cheng Lian Ee, Mohd Aspollah Sukari, Maizatulakmal Yahayu, Muhamad Aizat Mohd Amin, Abd Manaf Ali, Rusea Go

**Affiliations:** 1 Department of Chemistry, Universiti Putra Malaysia, 43400 UPM, Serdang, Selangor, Malaysia; 2 Department of Pharmacy, University of Malaya, 50603 Kuala Lumpur, Malaysia; 3 Faculty of Agriculture and Biotechnology, Universiti Sultan Zainal Abidin, 20400 Kuala Terengganu, Malaysia; 4 Department of Biology, Universiti Putra Malaysia, 43400 UPM, Serdang, Selangor, Malaysia

**Keywords:** antimicrobial, antioxidant, antiproliferative, *Artocarpus obtusus*, pyranocycloartobiloxanthone A, tyrosinase inhibitory

## Abstract

One of the most promising plants in biological screening test results of thirteen *Artocarpus* species was *Artocarpus obtusus* FM Jarrett and detailed phytochemical investigation of powdered dried bark of the plant has led to the isolation and identification of three xanthones; pyranocycloartobiloxanthone A (**1**), dihydroartoindonesianin C (**2**) and pyranocycloartobiloxanthone B (**3**). These compounds were screened for antioxidant, antimicrobial and tyrosinase inhibitory activities. Pyranocycloartobiloxanthone A (**1**) exhibited a strong free radical scavenger towards DPPH free radicals with IC_50_ value of 2 µg/mL with prominent discoloration observed in comparison with standard ascorbic acid, α-tocopherol and quercetin, The compound also exhibited antibacterial activity against methicillin resistant *Staphylococcus aureus* (ATCC3359) and *Bacillus subtilis* (clinically isolated) with inhibition zone of 20 and 12 mm, respectively. However the other two xanthones were found to be inactive. For the tyrosinase inhibitory activity, again compound (1) displayed strong activity comparable with the standard kojic acid.

## 1. Introduction

Interests in using natural products as starting materials for drug discovery has benefitted many therapeutic areas especially infectious diseases and oncology. It was estimated that approximately 75% of drugs for infectious diseases and 60% of anticancer compounds are either from natural products or their derivatives [1]. Plants, as well as other natural sources offer many important and active natural products which may differ in structure and biological properties. For example, phenolic compounds are one of potential components in plants well known for displaying many antioxidants activities especially in prevention of oxidative damage associated with many chronic diseases and pathological process including cancer and cardiovascular diseases as well as aging in living organism [2,3]. The changing pattern of diseases, numerous adverse effects with the current therapy and the emergence of bacterial strains resistant to many currently used antibiotics necessitates the exploration of our natural resources especially tropical plants that may give us great chances of discovering potential compounds.

Tropical plants may present a rich source of such compounds from which novel antibacterial and antifungal, antioxidants and chemotherapeutic agents may be obtained which can potentially be developed as alternative promising agents. Hence, many tropical plants from Malaysia are being and has been extensively studied as well as their biological activity. One of them is the *Artocarpus* species of the family Moraceae which consists of about 55 species and is widely distributed throughout subtropical and tropical regions of the World from Indian subcontinent south of the Himalayas, Sri Lanka, Burma, Thailand, Indo-China, Southern China, Taiwan and Malay Peninsula [4]. In Malaysia, especially Peninsular Malaysia itself, *Artocarpus* is represented by 20 species [5] and 23 (and one incompletely known) species are known in Sabah and Sarawak [4]. Many of the species are cultivated for their edible fruits such as *Artocarpus communis* or *altilis* (locally known as “sukun”), *A. heterophyllus* (“nangka”) and *A. integer* (“chempedak”) and some of the species are used for light construction materials and furniture due to their strong and durable dark-coloured wood [4,6]. Some parts of the plants are also used in traditional medicine preparations for the treatment of various diseases. The bark, leaves, seeds, fruits and roots of some species have medicinal properties and used as traditional medicine in Southeast Asia for the treatment of diarrhea, fever, liver cirrhosis, hypertension, diabetes, inflammation, malaria, ulcers, wound and for tapeworm infection [7,8,9,10,11]. Previous work on the constituents of the genus has led to identification of many interesting compounds especially phenolics, such as flavanones, flavones, stilbene, chalcones and xanthones [5,6,10,12]. Many interesting biological activities were also investigated and reported from previous phytochemical work on plant extracts and pure compounds isolated from *Artocarpus* species [5,6,7,9,13,14].

**Figure 1:**
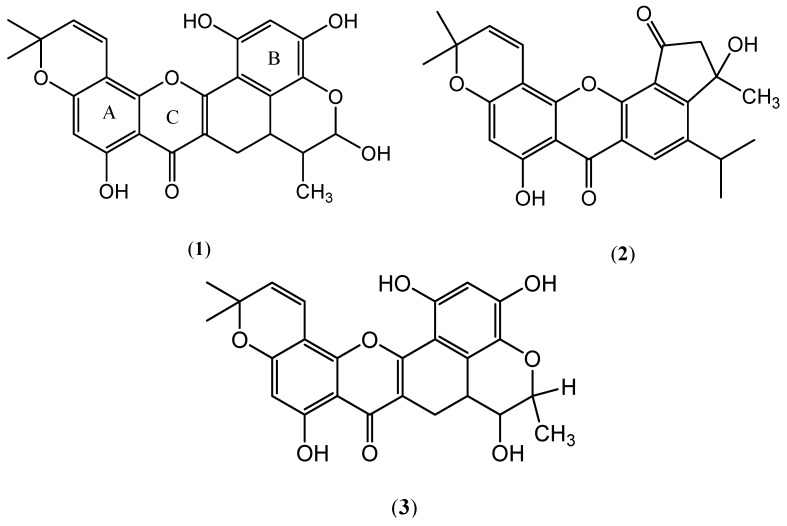
Chemical structures of pyranocycloartobiloxanthone A (**1**), dihydroartoindonesianin C (**2**) and pyranocycloartobiloxanthone B (**3**).

In previous communications we have reported the screening of thirteen *Artocarpus* species for biological activity and one of the plants, *Artocarpus obtusus*, was chosen for further detailed phytochemical and biological evaluation [15]. After extensive chromagraphic separation of the extracts, three new xanthones: pyranocycloartobiloxanthone A (**1**), dihydroartoindonesianin C (**2**) and pyranocycloartobiloxanthone B (**3**) (Figure 1) were isolated and structurally identified by spectroscopic techniques [16,17]. The compounds were further screened for antiproliferative activity against cancer cell lines and pyranocycloartobiloxanthone A (**1**) showed potent antiproliferative activity towards various cell lines and was inactive when tested on normal cell lines. Compound (**1**) was able to induce apoptosis against HL60 and MCF7 cell lines at it respective IC_50_ values. In continuation of our work on bioactive compounds from local plants [18,19,20], in this report we wish to further highlight the results of biological activity of the three xanthones when tested for antioxidant, antimicrobial and tyrosinase inhibitory activities.

## 2. Results and Discussion

### 2.1. Antioxidant Activity

**Figure 2:**
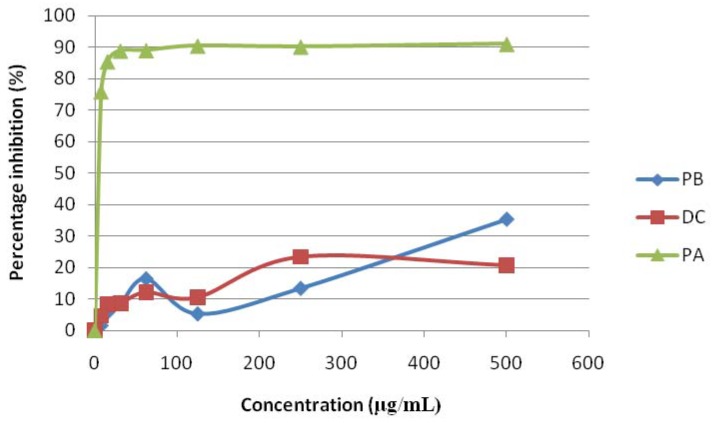
Percentage inhibition of DPPH (free radical) treated with different concentration of pyranocycloartobiloxanthone A (**1**) (PA), dihydroartoindonesianin C (**2**) (DC) and pyranocycloartobiloxanthone B (**3**) (PB) measured after 30 minutes using DPPH assay.

Pyranocycloartobiloxanthone A (**1**) demonstrated a strong free radical scavenger towards DPPH free radicals with IC_50_ 2 μg/mL obtained from the non-linear graph plotted (Figure 2). The prominent discoloration of DPPH after treatment with the compound from purple to yellow also suggested strong antioxidant effects. The IC_50_ value for the compound was found much lower than ascorbic acid (20 µg/mL), α-tocopherol (60 µg/mL) and quercetin (40 µg/mL) which indicated the compound possessed a strong free radical scavenger (Table 1). Both compounds dihydroartoindonesianin C (**2**) and pyranocycloartobiloxanthone B (**3**) showed very weak activity with IC_50_ values of more than 500 µg/mL (Table 1).

Previous studies on several prenylated flavonoid from *Artocarpus* species reported significant DPPH free radical scavenging activity [21]. Flavonoids are well known as hydrogen-donating antioxidants and react effectively as radical scavengers by donating hydrogen from hydroxyl groups to form resonance-stabilized phenoxy radicals [22]. Flavonoids are also known as chain-breaking antioxidants since their phenoxy radicals are sufficiently stable to inhibit chain propagating reactions (such as lipid peroxidation) [23,24]. Lipid peroxidation can cause cellular damages which can lead to several diseases such as arteriosclerosis, diabetes mellitus, neurodegenerative diseases associated with aging and carcinogenesis [25]. Evidence from previous *in vitro* studies showed that anthocyanins (flavonoid derivatives) possessed strong antioxidant property and are capable of scavenging all reactive oxygen and nitrogen species at least up to four times greater than ascorbic acid and α-tocopherol [26].

The effects displayed against DPPH radical suggested that these compounds were able to neutralize the radicals formed during the peroxidation. The presence of catechol group at the C-3′ and C-4′ positions of flavone skeletons and the phenolics containing three or two adjacent hydroxyl group are the major structural considerations underlying antioxidant activity [27]. Another factor that contributes to the activity is the hydrophobic properties, whereby with the presence of lipophilic substitution in the molecule, the hydrophobicity will increase and help to enhance the affinity of the compound for the plasma membrane [25]. An example is the α-tocopherol, an excellent chain breaking antioxidant, is the major lipid soluble antioxidant, whereby the lipophilic part is important for proper orientation of the molecule in the membrane [28]. Thus, the profound antioxidant activity exhibited by pyranocycloartobiloxanthone A (**1**), a flavone derived xanthone, possibly due to the presence of 2′,4′- dihydroxy substituents, hemiacetal ring and C3 substituted. This strong electron-donating groups (OH groups) would stabilize the free radical (DPPH free radical), and then inhibit the lipid peroxidation. 

### 2.2. Antimicrobial Activity

The compounds were evaluated for the antimicrobial activity using disc diffusion method and tested against eight bacterial and three fungal strains with streptomycin sulfate, gentamicin sulfate and nystatin used as positive controls in the assay. The results indicated that pyranocyloartobiloxanthone A (**1**) exhibited a broad spectrum activity against the microbes, whereas, the other two compounds were found to be inactive (Table 2).

The compound displayed strong and moderate antimicrobial activity against MRSA and *Bacillus subtilis* (clinically isolated strain) with inhibition zones of 20 and 12 mm, respectively. A weak antimicrobial activity was observed for the compound against *Bacillus subtillis* ATCC 6633, *Escherichia coli* ATCC 25922, *Staphylococcus aureus* ATCC 6538 and *Salmonella typhimurium* S865B with inhibition zones of less than 10 mm. However, *Micrococcus luteus* ATCC 10240 and *Pseudomonas aeruginosa* JCM 2412 were not susceptible to any of the isolated compounds. It was noted that, pyranocyloartobiloxanthone A (**1**) was a weak antifungal agent against the targeted fungi, *Candida albicans* ATCC 1023 and *Saccharomyces cerevisiae* S617 with inhibition zones of 7 and 8 mm, respectively. However, *Aspergillus niger* ATCC 16404 was not susceptible to the compound. The other two compounds **2** and **3** were not toxic towards any of the tested pathogenic fungi.

Two isoprenyl flavones, artocarpin and artocarpesin, obtained from the methanol extracts of *Artocarpus heterophyllus* strongly inhibited the growth of cariogenic bacteria, plaque-forming *Streptococci*. Similarly another prenylated flavonoid from *Artocarpus rigida* Blume also exhibited strong antimicrobial activity against *Escherichia coli* and *Bacillus subtilis* [29]. There were several studies carried out on structural-activity relationship among flavonoids based on the growth inhibitory zone by a paper disc method. The studies revealed that polyhydroxyl groups on the A and B rings (5-hydroxylation is essential) plus aliphatic substitution on the A ring are determinants for the antibacterial activity of the flavones [30]. In our study, only pyranocyloartobiloxanthone A (**1**) displayed a broad spectrum antimicrobial activity and this could be due to the presence of structural requirements as outlined above, the presence 5,2′,4′-trihydroxy substitutions in ring A and B. In contrast with compound **1**, the other two, **2** and **3**, did not render antimicrobial activity in spite of having the polyhydroxyl groups on the A and B rings in their chemical structure. This may be due to the steric hindrance of molecule in the compounds [31].

### 2.3. Tyrosinase Inhibitory Activity

In tyrosinase inhibitory assay, only pyranocycloartobiloxanthone A (**1**) was tested due to inadequate amounts of the other two samples. The results showed that pyranocycloartobiloxanthone A (**1**) exhibited a strong inhibitory activity against mushroom tyrosinase with an percentage inhibition of 80% (Table 1). The activity was comparable with kojic acid used as a positive control with a percentage inhibition of 96%. Previous work from *Artocarpus gomezianus* has resulted in isolation of potential tyrosinase inhibitors, a dimeric stilbene and flavonoid compounds [32]. Another study on tyrosinase inhibition activity from *Artocarpus incisus* has yielded several flavonoids, stilbenes and related 4-substituted resorcinols. Interestingly, the isolated compounds which displayed potent tyrosinase inhibitory activity had 4-substituted resorcinol as a common skeleton. The study on structure-activity relationship revealed that the 4-substituted resorcinol and C3 substituent is important criteria for the tyrosinase inhibitory activity [31]. The current study also revealed pyranocycloartobiloxanthone A (**1**) as a potent tyrosinase inhibitor against tyrosinase mushroom. The activity is again supported by structure-activity relationships of the related phenolics with the presence of 4-substituted resorcinol and C3 substituent as outlined above. 

From the above data obtained, pyranocyloartobiloxanthone A (**1**) displayed a great potential and promising activity as an antioxidant, a broad spectrum antibacterial agent and tyrosinase inhibitor. The chemical structure of the compound may contribute to the significant activity observed for the compound. Amazingly, all three compounds possessed the resorcinol moieties in ring B and substituents at C-3 or polyhydroxyl substituents in ring A and B which have been proposed previously to associate with significant activity against selected assays [6,31,33,34]. However, their activities toward the selected assays varied remarkably. Therefore, we can conclude that, despite having all the structural requirements, other additional factors are necessary to establish the activity such as less bulky substituents, the number and positioning of the hydroxyl group and the presence of prenyl (C_5_) groups which varies among the phenolics. The above findings are believed to be the first study or reports from the isolated compounds.

## 3. Experimental

### 3.1. Plant Materials

Sample of the air dried stem bark of *Artocarpus obtusus* was collected from Sarawak in 2004, identified by Dr. Rusea Go and a voucher specimen (S94402) has been deposited at the Herbarium, Department of Biology, Faculty of Science, Universiti Putra Malaysia. 

### 3.2. Chemicals and Reagents

1,1-Diphenyl-1-picrylhydrazyl (DPPH) reagent (Sigma, USA), ascorbic acid (Sigma, USA), chloroform (analytical grade, Merck), l-DOPA (Sigma), dimethyl sulphoxide (DMSO) (analytical grade, Merck), gentamicin sulfate 10 μg/disc (Oxoid), *n*-hexane (analytical grade, Merck), kojic acid (Sigma), methanol (analytical grade, Merck), mushroom tyrosinase (Oxoid), nutrient agar (Oxoid), nutrient broth (Oxoid), nystatin 100 IU/disc (Oxoid), potato dextrose agar (Oxoid), potato dextrose broth (Oxoid), phosphate buffer (Sigma), quercetin (Sigma, USA), streptomycin 10 μg/disc (Oxoid) and α-tocopherol (Sigma, USA).

### 3.3. Preparation of Plant Extracts and Isolation Procedure

The dried ground stem bark (640 g) of *Artocarpus obtusus* was sequentially extracted with *n*-hexane, chloroform and methanol at room temperature. The isolation procedure and identification of the compounds has been described in our earlier reports [19,20].

### 3.4. DPPH Free Radical Scavenging Activity Assay

The standard DPPH assay was used with slight modification. In this the technique, 96 micro-well flat bottom plates were used instead of cuvettes to consume minimal amounts of sample, vehicle and DPPH solutions. Stock solutions of the compounds were prepared at 1 mg/mL in DMSO. Each well was filled in with 100 μL of diluent (DMSO). The first row (row A) of the 96 wells was filled in with 100 μL of diluted sample at 1 mg/mL. Concentration in the first row was 500 μg/mL of sample in each well and was serially diluted (two fold dilution) in 96 micro-well plates to varying concentrations, topping from 500 μg/mL down to the lowest 7.8 μg/mL. Then, 5 μL of the DPPH solution (2.5 mg/mL in DMSO) was added to each well. The DPPH solution should be kept in the dark at 4 °C. This procedure should not be done under direct light. The plate was shaken to ensure thorough mixing before placed in the dark. After 30 minutes, the optical density of each well was read using ELISA Reader (EL340 Biokinetic Reader, Bio-Tek Instrumentation) at wavelength 517 nm. Percentage inhibition was calculated using the following formula:

% Inhibition = 1 − OD(DPPH + Sample)/OD(DPPH) × 100

A graph of percentage inhibition of free radical activity was plotted against concentration of crude extract and 50% inhibition concentration (IC_50_) can be obtained from the graph. The IC_50_ value was determined as the concentration of each sample required or able to scavenge 50% of the DPPH. All tests and analyses were run in triplicate and averaged. Antioxidant assay by using DPPH method was used to assess the capacity of the three compounds to scavenge stable free radical DPPH at different concentration. The radical scavenging effect was examined and compared with natural antioxidants a-tocopherol (Sigma, USA), ascorbic acid (Sigma USA), and quercetin (Sigma, USA) used as positive controls (Table 1).

### 3.5. Bacterial and Fungal Cultures

The bacterial strains employed in the screening were *Bacillus subtilis* (clinically isolated strain), *Bacillus subtilis* ATCC 6633, *Escherichia coli* ATCC 25922, Methicillin resistant *Staphylococcus aureus* (MRSA) (ATCC 33591), *Micrococcus luteus* ATCC 10240, *Pseudomonas aeruginosa* (JCM 2412), *Salmonella typhimurium* S865B (IMR culture) and *Staphylococcus aureus* ATCC 6538. For the antifungal test, the strains employed were *Aspergillus niger* ATCC 16404, *Candida albican* ATCC 1023 and *Saccharomyces cerevisiae* S617 (IMR culture).

### 3.6. Disc Diffusion Assay

Antimicrobial activity of the crude extracts was determined by the disc diffusion method [35] with slight modification in term of sample concentration, volume of sample loaded and use of paper discs. The bacteria and fungi were cultured at 37 °C for overnight in nutrient broth and potato dextrose broth, respectively. The concentrations of the cultures were adjusted turbidometrically at a wavelength of 600 nm which give 10^5^–10^6^ colony forming units (CFU) per mL. The compounds to be tested were dissolved in dimethyl sulphoxide (DMSO) at concentration of 1 mg/mL. For the purpose of screening, 10 μL of each sample solution was loaded on Whatman No.1 filter paper disc (∅ 6 mm). The disc was placed on the surface of the agar plate (nutrient agar or potato dextrose agar) previously inoculated with bacteria. The agar plates were then inverted and incubated for 24 h at 37 °C. The antimicrobial activity was recorded by measuring the zone of inhibition (in mm) around each disc. Each test was carried out in triplicate. Antibiotics streptomycin sulfate (10 μg/disc), gentamicin sulfate (10 μg/disc) and antifungal nystatin (100 IU/disc) were used as positive control and DMSO as negative control in the assays.

### 3.7. Tyrosinase Inhibitory Activity Assay

Tyrosinase inhibitory activity was assayed by using the modified dopachrome method with modifications [31] with the concentration of sample used was 1 mg/mL. l-DOPA was used as the substrate and mushroom tyrosinase as the enzyme. The assays were conducted in 96-well microtiter plate, and μQuant Biotek reader was used to measure the absorbance at 475 nm. For each concentration of the sample solution, four wells designated A, B, C and D each contained a reaction mixture (180 μL) as follows:

(A) 20 μL of mushroom tyrosinase in 20 mM phosphate buffer (480 units/mL) Sigma
(B) 140 μL of 20 mM phosphate buffer (pH 6.8), and 20 μL of methanol
(C) 20 μL of mushroom tyrosinase solution (480 units/mL), 140 μL of 20 mM phosphate buffer (pH 6.8), and 20 μL of sample solution
(D) 160 μL of 20 mM phosphate buffer (ph 6.8) and 20 μL of sample solution.

Each well was mixed and incubated at 25 °C for 10 min. Then, 20 μL of 0.85 mM l-DOPA (Sigma) in phosphate buffer (pH 6.8) was added. After incubation at 25 °C for 20 min, the amount of dopachrome in each reaction mixture was measured as the difference of the optical density before and after incubation. The percent inhibition of tyrosinase activity was calculated using the equation below:

% Inhibition = 100[(A-B) − (C-D)]/(A-B)

where; A represents the difference of optical density before and after incubation without test sample; B represents the difference of optical density before and after incubation without test sample and without enzyme; C represents the difference of optical density before and after incubation with test sample; D represents the difference of optical density and after incubation with test sample and without enzyme. Kojic acid (Sigma) was used as positive standard.

## 4. Conclusions

The three xanthones isolated and identified from *Artocarpus obtusus* were evaluated for their free radical scavenger, antimicrobial and tyrosinase inhibitory activities. The results showed that pyranocycloartobiloxanthone A (**1**) possessed a potent free radical scavenger with an IC_50_ value of 2 μg/mL and showed strong and moderate antimicrobial activity against MRSA and *Bacillus subtilis* (clinically isolated strain) with inhibition zones of 20 and 12 mm, respectively. However, compound (**1**) showed weak activity against *Bacillus subtillis* ATCC 6633, *Escherichia coli* ATCC 25922, *Staphylococcus aureus* ATCC 6538 and *Salmonella typhimurium* S865B. The potent free radical scavenging activity displayed from compound (**1**) signifies that the compound possesses strong antioxidant properties and may potentially become a promising alternative antioxidant in the future and thus may provide great benefits to therapeutic areas especially cancer. The significant antimicrobial activity of compound (**1**) revealed that the compound displayed a broad spectrum activity against Gram negative and Gram positive bacteria. The compound also exhibited a good tyrosinase inhibitor activity with 80% of inhibition which may potentially influence the melanin biosynthesis in plants, microorganisms and mammalian cells. The varied activity of pyranocycloartobiloxanthone A (**1**) may benefit from its chemical structure. However, the results of selected bioassays on the other two xanthones were not as pronounced. In a nutshell, the presence of a 4-substituted resorcinol moiety in ring B, hemiacetal ring, with or without an isoprenyl substituent at C-3, less bulky substituents, the number and positioning of the hydroxyl group may implicated different activity from the three compounds in each assay.

## Figures and Tables

**Table 1:** The IC_50_ values of the pyranocycloartobiloxanthone A (**1**), dihydroartoindonesianin C (**2**) and pyranocycloartobiloxanthone B (**3**) against DPPH (free radical) and percentage inhibition of tyrosinase.

Compounds	IC_50_ (μg/mL)	% Inhibition
Pyranocycloartobiloxanthone A (**1**)	2 ± 1.2	80
Dihydroartoindonesianin C (**2**)	>500	not tested
Pyranocycloartobiloxanthone B (**3**)	>500	not tested
		
Standard		
Ascorbic acid	20 ± 1.2	
α-tocopherol	60 ± 2.9	
Quercetin	40 ± 5.8	
Kojic Acid		96

Data represent mean ± SD of triplicate determinations from three independent experiments.

**Table 2:** The inhibition zone diameter (in mm) of pyranocycloartobiloxanthone A (**1**), dihydroartoindonesianin C (**2**) and pyranocycloartobiloxanthone B (**3**) against pathogenic microbes.

Microorganisms	Inhibition zone diameter (in mm)					
	(1)	(2)	(3)	Gentamicin sulfate	Streptomycin sulfate	Nystatin
Bacteria						
Reference strains						
Bacillus subtilis ATCC6633	8	-	-	15	nt	
*Escherichia coli* ATCC25922	7	-	-	25	nt	
*Micrococcus luteus* ATCC10240	-	-	-	nt	nt	
Methicillin resistant *Staphylococcus aureus* ATCC33591	20	-	-	nt	20	
*Pseudomonas aeruginosa* JCM2412	-	nt	nt	15	21	
*Staphylococcus aureus* ATCC6538	9	-	-	11	nt	
*Salmonella typhimurium* S865B (IMR culture)	9	-	-	nt	nt	
						
Clinically isolated strain *Bacillus subtilis*	12	nt	nt	nt	-	
Fungi						
Reference strains						
*Aspergillus niger* ATCC16404	-	-	-			nt
*Candida albican* ATCC1023	7	-	-			15
*Saccharomyces cerevisiae* S617 (IMR culture)	8	-	-			23

Note: (-) – not active, (nt) – not tested. Streptomycin sulfate 10 μg/disc, gentamicin sulfate 10 μg/disc, nystatin 100 IU/disc; pyranocycloartobiloxanthone A (**1**), dihydroartoindonesianin C (**2**), pyranocycloartobiloxanthone B (**3**).
